# Feasibility and Impact of Community Pharmacy and Novel Pick-up Points for Antiretroviral Therapy Pre-exposure Prophylaxis Initiation and Continuation in Low and Middle-income Countries

**DOI:** 10.1007/s11904-024-00710-3

**Published:** 2024-11-16

**Authors:** Samanta Tresha Lalla-Edward, Willem Daniel Francois Venter

**Affiliations:** Ezintsha, Faculty of Health Sciences, University of the Witwatersrand, Sunnyside Office Park, 32 Princess of Wales Terrace, Johannesburg, South Africa

**Keywords:** HIV Prevention Treatment, Community Pharmacist, Differentiated Service Delivery, Retention in Care

## Abstract

**Purpose of Review:**

This review assesses recent developments in community access to pre-exposure prophylaxis (PrEP) for HIV prevention in low-and middle-income countries (LMICs). It examines literature on differentiated service delivery (DSD) and alternative delivery modes for PrEP, focusing on the role of community pharmacies and novel pick-up points. Key considerations include barriers to access, potential benefits, and strategies for implementation.

**Recent Findings:**

Challenges to optimal HIV healthcare delivery persist globally, with LMICs facing greater barriers due to resource constraints and structural obstacles. Community pharmacies and novel pick-up points offer promising avenues to expand access to HIV medication, especially in hard-to-reach populations. However, operational complexities and regulatory frameworks present significant challenges. Recent initiatives, such as collaborative practice agreements and programmes by global health agencies, highlight efforts to integrate community pharmacies into HIV prevention and care delivery. Mobile health clinics and home delivery services have also shown promise in improving treatment coverage.

**Summary:**

Community pharmacies and novel pick-up points play a crucial role in enhancing access to HIV PrEP in LMICs. Despite challenges related to infrastructure, funding, and regulatory oversight, innovative strategies like DSD and mobile outreach offer opportunities to reach marginalized populations. Real-life examples from LMICs demonstrate the feasibility and effectiveness of leveraging community pharmacies for HIV treatment. However, addressing policy gaps, strengthening pharmacist training, and promoting patient-centred approaches are essential for scaling up access to PrEP. Collaboration between governments, health agencies, and local communities is key to realizing the full potential of community pharmacies in HIV prevention and care.

## Introduction

Among its recommendations for delivering optimal HIV healthcare, the World Health Organization (WHO) emphasises differentiated service delivery (DSD) for HIV treatment in its *“Consolidated HIV guidelines for prevention, treatment service delivery & monitoring”* (2021) [1]. This is particularly true for low- and middle-income countries (LMICs), where various challenges prevent these life-saving medications from reaching those who need them. Traditional healthcare facilities, like hospitals and clinics, often are inadequate in providing sufficient coverage, and are inaccessible to certain populations [2]. With such challenges, community pharmacies and novel pick-up points may provide greater access to antiretroviral therapy (ART) and pre-exposure prophylaxis (PrEP) [3–5].

Barriers to optimal healthcare occur globally, and while lags in service provision are common in both high-income countries as well as LMICs, the delays are more frequent and severe in the latter [6]. Some of the most common clinical barriers to implementing evidence-based practices in LMICs are shortages of clinical equipment and facilities as well as insufficient staffing [7]. Compounding the problem is that some communities experience additional challenges driven by their socio-economic contexts. For example, one investigation into determinants of healthcare access for women in 36 sub-Saharan countries, found that factors like distance from healthcare facilities, transport service availability and cost factors amongst others, influenced the extent to which they were able to access healthcare [8]. These barriers can also be extrapolated to patients’ access and willingness to commence HIV care in resource constrained settings [9, 10].

PrEP access has been well demonstrated within the USA, and there is general interest in expanding access to services within pharmacies within high, middle- and low-income arenas. These may include acute interventions like collaborative practice agreements, where pharmacists after entering into a collaborative-practice agreement with primary care clinics are permitted to provide PrEP [11–13]. Further, programmes driven by The Global Fund and US President’s Emergency Plan for AIDS Relief (PEPFAR) for example, have prioritised alternative drug collection points as part of DSD [14, 15].

There have been calls within high income countries and LMICs alike for community pharmacies to be involved in the provision of HIV medication. They are highly accessible, since community pharmacies obviate the requirement for HIV treatment seekers to have an appointment to visit a health practitioner to access their medication. Moreover, they are also able to stave off HIV related stigma, and community pharmacists are trained healthcare professionals. However, significant operational complexity accompanies LMIC programmes, where donors and governments are usually responsible for procuring and supplying PrEP. For community pharmacists to be fully utilised in the role of suppliers and administrators of HIV medicines, governments need to authorise and oversee community pharmacies as drug roll-out avenues, healthcare governing bodies need to formulate rules by which community pharmacies must abide, strategies to improve infrastructure and funding provision must be in place, and specialised training programmes for pharmacists must be developed [16–18].

Providing access to HIV treatment requires an understanding of what drives non-adherence. Meka et al. [9] and Muhumura et al. (2021) found that various fears, interpersonal and community factors, as well as costs and concerns regarding healthcare facility personnel and processes, were the most significant barriers [9, 19]. A closer look at how community pharmacies in Asia and United States are utilised, can shed additional light on the opportunities they present to enhance access to HIV services. The value of DSD was confirmed during the COVID-19 pandemic, where an investigation into pharmaceutical care services revealed that at the height of the pandemic, in the Philippines, the public relied on community pharmacies for acquiring personal protective gear [20]. Furthermore, community pharmacists in India and the United States, are increasingly being embraced as the providers of personalised care to patients as well managing non-communicable diseases [21, 22]. These examples suggest that community pharmacies can move beyond their focus of dispensing medication, to providing extended individualised HIV services. Having said that, this stands a greater chance of success when executed in a culture-sensitive manner. In countries like Thailand where the transition away from traditional medicines is in its developmental stages, and restrictive guidelines for pharmacies are prevalent, the adoption of community pharmacies has received little recognition [23]. These are important considerations when scrutinising the potential contributions of community pharmacies in any HIV related prevention and management.

In this review, we assess recent developments surrounding community access to PrEP, focusing on literature addressing DSD or alternative modes of delivery of PrEP (including non-conventional clinic/hospital-based initiation). Additional to search engines we reviewed critical LMIC guidance documents from key agencies, including WHO, as well as guidelines from major LMIC countries. We paid particular attention to countries such as Kenya and South Africa, where significant work within pharmacies had been profiled, and countries where PEPFAR had made progress with DSD. What this review explores is the extent to which community pharmacies and novel pick-up points in LMICs support access to ART PrEP, the drivers of and barriers to their provision of HIV medication, as well as the potential for improvement in this arena.

## Pharmacy Structure

Overall, there is an insufficient number of pharmacies in Africa. While most pharmacists are employed in the community pharmacies in private health in almost all of Africa, in Kenya approximately 70% are employed in hospitals [24]. Most pharmacists opt to work in urban rather than rural locations. South Africa having one of the most substantial pharmacy personnel, also offers a variety of pharmacy locations and it is not uncommon to find licenced retail community pharmacies in shopping malls or residential areas [25]. Chain pharmacies like DisChem have garnered prominence in South Africa, as have pharmacies located in popular hypermarkets, like Clicks. Such pharmacies, regulated by the South African Pharmacy Council, are typically owned and operated by licenced pharmacists [26].

The biggest drawcards of community pharmacies are that they are convenient given that they service the particular pharmaceutical and/or healthcare needs of a certain community or even tailor-made ones for certain individuals within that community. In addition, they provide a range of services that go beyond dispensing medication, like providing healthcare guidance and health screening [27, 28]. The goal of these pharmacies is to promote improved healthcare outcomes for the community by offering convenient and accessible services [26]. This type of DSD is one way in which to increase access to HIV medication. The WHO clarifies the role that innovative interventions can play in boosting the expansion of HIV services. These include the use of fixed pick-up points like community pharmacies as well as other novel pick-up points like mobile outreach ART delivery [29].

## Novel Pick-up Points

To facilitate access to PrEP, numerous projects have been undertaken in the hope of increasing its uptake with regards to both initiation and adherence. Reflecting on programmes geared to adolescent girls and young women indicate that the demand for PrEP often wanes after enrolment. Some of the reasons for this loss of interest have been attributed to inadequate support for users, inconvenience and complexity in accessing PrEP, and the frequency the medication needed to be taken [30, 31]. To combat these barriers, various solutions were proposed. In Kenya, pharmacy delivery of PrEP to adolescent girls and young women was tested as one of the reach mechanisms for this population; found that it was a preferred delivery mode, and there was willingness to pay for accessibility, confidentiality, and high quality of services [32].

Likewise, novel pick-up points may reach key populations living with HIV or who are at risk of contracting HIV, and who are often marginalised. Traditionally, these are sex workers, men who have sex with men, the transgender community, and injecting drug users [33]. The Tanzanian FIKIA project concluded that when a client centred approach which is tailored to provide bespoke HIV services to a particular marginalised population is adopted, the outlook for the testing and prevention or treatment of HIV is promising indeed [34].

DSD strategies can achieve improved HIV prevention and treatment coverage. Apart from attempting to accelerate initiation on HIV medication, it has been proposed that DSD could possibly reduce patient traffic in health facilities while addressing other structural obstacles to the adequate provision of HIV treatment. Extrapolating to prevention, novel pick-up points include the use of community based ART and PrEP delivery methods, mobile clinics, tents and delivering medicines to patients’ homes [35].

The use of mobile health clinics to provide health services for a number of diseases has been documented: in Brazil and Thailand it has been used for mother and child health [36, 37], in India for diabetes [38], and in Namibia, primary healthcare for children [39]. All these studies were conducted on at risk people in rural or underprivileged populations, where typically medical interventions were irregular. In every study, larger numbers of people were reached, and in the Brazilian and Asian studies there was a significant improvement in the management of disease. In South Africa, the Tutu Teen Truck (TTT) initiative was undertaken, whose target is delivering sexual health treatment for adolescent girls and young women from 16–25 years of age. Over the span of just over two years, 585 young women initiated PrEP medication [40]. This initiative received praise from those who accessed their services, as TTT provided them with information that they felt too embarrassed to enquire about normally, and because they were able to access PrEP in confidence.

During the COVID-19 pandemic, implementing the use of novel pick-up points for the distribution of HIV PrEP became an urgency, when governments needed to re-strategize on how to continue delivering PrEP to maintain the momentum of HIV prevention. Two strategies worked particularly well in Uganda. One was medication delivery by motorcyclists, where healthcare providers would contact motorcyclists who would then deliver medication to patients’ homes. This had the benefit of not only circumventing the transport and movement restrictions unique to the pandemic context, but also meant that patients who experienced problems accessing healthcare facilities would still be able to get their refills timeously. In addition, community pharmacies dispensed HIV medication that would cover multiple months, reducing the need for accessing distribution facilities monthly. Community pharmacies, because they were already located in patients’ immediate vicinity, meant that patients could have their HIV medication refilled at their convenience [41]. However, there are also instances where the success of delivering ART onsite was short-lived, like Sisters Antiretroviral Therapy Programme for Prevention of HIV in Zimbabwe. When PrEP availability was decentralised to community facilities to accommodate for COVID-19 pandemic’s movement restriction, the demand for PrEP increased from 315 monthly (pre-pandemic) to a high of 3084 from July to September 2020 (during the pandemic). Stock shortages forced sites to either restrict or completely stop new PrEP initiations, and by December 2020, the number reduced to 1548 [42]. While it is unfortunate that a successful programme could not reach its full potential, the lesson to be learned is that DSD can facilitate an uptake in PrEP.

Another interesting solution to increasing the uptake of HIV medication, is combining the use of digital technology or telemedicine with other novel pick-up point ones. Brazil, during COVID-19 implemented a system where patients could request HIV rapid diagnostic tests or PrEP prescriptions telephonically, could send in digital pictures of their results if they had just tested, and would receive a digital prescription or home delivery aligned to their HIV requirements. Slight variations of this programme also operate in Vietnam and Kenya among others. This multipronged intervention had a high acceptability amongst users [10].

## Potential for PrEP

Evidence from high income countries show that using community-based pharmacies in collaboration with the health department to deliver PrEP, and even for PrEP follow up is viable, since both patients and pharmacists showed a keenness for this service. It may be one of the solutions to reaching rural populations in particular [43, 44]. Furthermore, when both community pharmacists and patients or participants displayed confidence in the pharmacists’ ability to deliver proper point of care intervention (including provision of emergency contraception, contraception, post exposure prophylaxis and HIV testing), the success rate of engaging patients in HIV interventions (including bundling of services e.g. offering PEP with emergency contraception or HIV testing with all sexual reproductive health services, or using vaccinations as an entry point) was encouraging [45, 46]. An Australian study also concluded that engaging community pharmacists who were self-assured in both their knowledge of HIV medicines and their ability to dispense the medication, could result in improved medicine adherence [47]. Researchers responsible for a ground-breaking study in Kenya conducted at five retail pharmacies, reasoned that it was viable to deliver PrEP services at pharmacies which had confidential counselling areas and where personnel were trained and willing to execute provider-assisted oral-fluid HIV testing. They based their conclusion on clients’ reports which indicated extremely high fidelity of PrEP services in counselling, maintaining client privacy, self-testing assistance, and PrEP dispensing [48].

## Acceptability of Pharmacies as PrEP Access Points

Various strategies have been used to introduce PrEP into pharmacies. Exploring them can help to identify which of them have a reasonable chance of success in implementation in resource strained contexts and under what conditions.

One solution to reduce the stresses associated with accessing HIV PrEP treatment particularly, is long acting (LA) injectables [49]. Recent studies on LA HIV drugs’ potential to address these challenges, conclude that while the alternative of using LA HIV medication is a viable option, “knowledge gaps” along with many other challenges pertaining to systemic contexts, as previously alluded to. Should these challenges not be met, the implementation of LA HIV therapy will be jeopardised [50, 51]. This suggests that reformulating HIV medication will not make a big enough impact in the prevention and treatment of HIV, if not implemented with tandem strategies to alleviate the non-medical barriers.

Generally speaking, HIV testing has improved [52], and testing provided by pharmacies is widely sought [53]. A study undertaken in southern Nigeria showed that not only were community pharmacists agreeable to providing HIV care, but patients were also willing to use these services. Even so, just 30 community pharmacies out of just over 370 offered HIV management services [54]. However, the agreeableness of pharmacists to provide treatment to people with HIV is not always positive, as revealed in a Malaysian study. The authors in their investigation found that while most pharmacists were unwilling to treat people with HIV, community pharmacists were less disagreeable [55]. They explain that the unwillingness stems from cultural beliefs and stigma associated with having HIV, and that this attitude has even been documented in other resource limited countries. However, pharmacists with more robust health knowledge have less bias against prevention and treatment HIV efforts, as was evidenced in South America [55].

## Real World Application

Early results from implementation projects undertaken in LMICs are encouraging, as exemplified in the Nigerian studies. A pilot study assessing the feasibility of using community pharmacies as pick-up points for HIV medication, found that every patient with HIV who accessed a community pharmacy, refilled their prescription, and 99.3% of them were retained in care [56]. Another conducted with people living with HIV being redirected to community pharmacies drew similar conclusions and established that patients were amenable to paying for the services accessed at the community pharmacies [57]. Furthermore, in one investigation the average CD4 cell count and weight of patients who accessed community pharmacy services also increased [58]. One Kenyan evaluation reported definite feasibility and acceptability of private pharmacy-based delivery of PrEP among populations not engaged in traditionally delivered health services [59] and another reported high numbers of adolescent girls and young women purchasing contraception starting on PrEP and expressing a willingness to pay for it [60]. A pharmacist led PrEP clinic in the United States demonstrated itself as a successful alternative approach to PrEP care, boasting high rates of initiation and a minimal number of people falling out of the programme [3].

Table 1 summarises selected studies and interventions mentioned in this review to this point.

**Table 1:** Summary of selected studies/interventions reported against implementation outcomes

Intervention/Study	Description/Method	Year of implementation/ review *	Setting (continent/region/ country) **	Reported results aligned to Proctor's implementation outcomes (acceptability; adoption; appropriateness; costs; feasibility; fidelity; penetration; sustainability) [61] or RE-AIM (reach; effectiveness; adoption; implementation; maintenance) [62] ***
A pharmacist-managed HIV PrEP clinic in a community pharmacy setting at Kelley-Ross Pharmacy in Seattle (3]	The clinic operated under a collaborative drug therapy agreement based on the 2014 US National guidelines for the provision of PrEP. Pharmacists were permitted to initiate and manage tenofovir disoproxil fumarate/emtricitabine under the supervision of a physician medical director	2015–2018	USA	**Adoption (Uptake)**: 695/714 (97.3%) initiated PrEP. 513/695 (74%) patients began medication at their initial appointment. Of the prescriptions filled in pharmacy, 90% of patients had a mean proportion of days covered greater than 80%. **Maintenance**: 19% of patients were lost to follow up (effective drop-out rate of 25%). 207 diagnoses of STIs were made. **Effectiveness**: There were no HIV seroconversions. **Implementation cost**: 98% had a zero-dollar patient responsibility per month, including uninsured individuals.
Systematic review of systematic reviews, meta-analyses, and narrative reviews using selected databases to assess the impact of community pharmacist-led interventions in chronic disease management on clinical, utilisation, and economic outcomes [4]	Extracted data included, citation details, review type, number of primary studies included, disease state, intervention description, outcomes assessed, and results. Fifteen references met the inclusion criteria. Selected articles covered seven main diseases —diabetes, asthma, chronic obstructive pulmonary disease, hypertension, heart failure, hyperlipidaemia, and HIV	2007–2017	Various	Community pharmacist-led interventions were patient consultations and education. **Effectiveness**: Diabetes interventions achieved significant reductions in haemoglobin A1c, total cholesterol, and low-density lipoprotein. Reductions in blood pressure, improved medication adherence and reduced readmission rates in patients with heart failure, improved lung function in patients with respiratory conditions, and increased medication adherence in those with HIV. **Implementation cost**: Economic and utilisation outcomes were limited—mainly pharmacy interventions in diabetes. In some cases, community pharmacy services demonstrated a decrease in medical and health care costs in patients with diabetes.
Five differentiated ART delivery models implemented at a national sample of health facilities to improve the quality of HIV care and to achieve health-system efficiencies [5]	Qualitative exploration of reasons for variations in uptake of facility-based versus community-based models. Community-based models included community client-led ART delivery and community drug distribution points. Facility-based models included fast track drug refill, facility-based group and facility based individual management	2019–2020	Uganda	**Adoption**: 63/116 (57%) facilities commenced implementation of differentiated ART delivery in 2018. **Implementation**: Fast track drug delivery was the most common facility-based model—100/116 (86%) health facilities. Community client-led ART delivery was the most popular community model—63/116 (54%). Community drug distribution points had the lowest uptake—33/116 (24.88%) facilities implementing them. By ownership-type, for-profit facilities (*n* = 21) reported the lowest uptake of differentiated ART models—33%. 67% of all public (*n* = 68) and 52% of all private not-for-profit (*n* = 27) commenced implementation of differentiated ART delivery models in 2018.
A review of technology-based interventions for HIV in LMICs to highlight potential benefits and challenges to using telehealth in LMICs and propose areas for further investigation [10]	Several models were identified for using telehealth to expand HIV health care access in LMICs, including telemedicine visits for PrEP and ART services	2008–2022	Various including Africa, Asia, Latin America, other	Notable examples of tele-PrEP implementation in LMICs: **Vietnam:** the Ministry of Health launched a national tele-PrEP pilot programme with the participation of 20 clinical sites in seven provinces. According to the Vietnam tele-PrEP guidelines, an initial in-person consultation is required for clinical examination, baseline laboratory test, and PrEP prescription. If the client is enroled in tele-PrEP, follow-up visits are conducted via telephone or video-conferencing consultation with a remote prescription for PrEP medications which can be obtained at the pharmacy or delivered to the home. HIV testing is recommended before each PrEP follow-up visit and must be conducted at a certified laboratory or via a home-based laboratory service. **Thailand:** the COVID-19 pandemic accelerated the roll-out of tele-PrEP within existing KP-led PrEP health services. PrEP is prescribed remotely by doctors and dispensed by KP lay providers. PrEP initiation and initial follow-up visits are in-person. Virtual visits and self-sampling and self-testing for HIV and other STIs were offered to those on PrEP for more than 3 months. A second fully online PrEP delivery model in Thailand provided comprehensive HIV services through telehealth including ART and PrEP services. For PrEP, if an individual had a negative HIV test within 7 days of the appointment, a creatinine within 6 months and a hepatitis B surface antigen test within 1 year, they could see a physician via video conferencing, make an on-line payment, and receive PrEP medications at home. **Kenya:** Telehealth services were integrated into the HIV prevention and PrEP programmes for AGYW. Outreach and counselling through “virtual safe spaces” combined with home delivery of PrEP medications, HIV self-test kits, sanitary packs, and condoms enabled continuity of prevention services. **Acceptability**: In **Brazil**, telehealth strategies were developed and implemented to maintain HIV PrEP services among adults and adolescents during COVID-19-related health system disruptions. A cross-sectional web-based survey of PrEP users demonstrated high acceptability with PrEP teleconsultation and home delivery.
A systematic review of studies examining effectiveness, values and preferences of end users and health workers, and cost of PrEP initiation and continuation through pharmacies (pharmacy access) [11]	This review addressed two related questions: whether PrEP initiation should happen in pharmacies, and whether PrEP continuation should happen in pharmacies. Review focussed on in-person pharmacy initiation and continuation and excluded telemedicine-based approaches. Literature in three areas relevant to answering these questions: effectiveness of the intervention, values and preferences of end users and health workers and cost information were assessed	inception—2020	Various including Kenya, South Africa, USA, Zimbabwe, other	**Effectiveness:** Zero studies met the inclusion criteria for the effectiveness for either PrEP initiation or continuation. **Feasibility**: Six case studies presenting non-comparative data from PrEP pharmacy programmes demonstrated feasibility of the model in the USA. **Acceptability**: Eleven studies reported values and preferences of end users and health workers. In the USA, Kenya and South Africa, potential PrEP clients generally supported pharmacy access, although some preferred clinics. One study of PrEP pharmacy clients found all would ‘definitely recommend’ the programme. Six studies found pharmacists were generally supportive of offering PrEP; one study including doctors found more limited favour, while one study of diverse Kenyan stakeholders found broad support. **Cost/implementation cost**: Three studies reported cost data indicating client willingness to pay in the USA and Kenya and initial sustainability of a clinic financial model in the USA.
A scoping review assessing pharmacy-based initiatives implemented to increase PrEP use [13]	Research activities, summarised findings, and consolidated recommendations in the literature concerning pharmacy-based PrEP interventions were examined in the PubMed, PsycINFO, CINAHL and Scopus databases. Forty-nine articles were eventually included	2012—2021	USA	**Acceptability**: Patients expressed strong support for pharmacist prescription of PrEP. Three intervention designs compared changes in PrEP initiation or knowledge pre- and post-intervention. **Feasibility**: Commentary/ review studies recommended PrEP training for pharmacists, policy changes to support pharmacist screening for HIV and PrEP prescription, and telemedicine to increase prescriptions.
A review to provide a global description of PrEP delivery using private sector pharmacies by discussing context-specific factors that have influenced the design and implementation of pharmacy-based PrEP delivery models in three settings, and to identify future research directions [18]	Three countries, each with over 300,000 cumulative PrEP initiations were assessed. In select parts of the USA, South Africa, and Kenya, three main pharmacy PrEP models were implemented programmatically and/or tested via research studies: (1) a co-located model (with no task shifting), (2) a partially task shifted model, and (3) a fully-task shifted (i.e., standalone) model	2015–2023	Kenya, South Africa, USA	**Acceptability**: Pharmacy-based PrEP services are in demand and acceptable to clients and pharmacy providers. **Feasibility**: With proper training and oversight, pharmacy providers are capable of safely initiating and managing clients on PrEP. **Adoption**: Pharmacies can increase overall PrEP coverage. The delivery of PrEP services at private pharmacies yields similar levels of PrEP initiation and continuation as traditional health clinics, but reach those underserved by clinics (e.g., young men). **Implementation:** Pharmacy-based PrEP services will be context specific and depend on the state of the private pharmacy sector, and the extent to which needs related to governance, financing, and regulation are addressed.
An evaluation the acceptability and feasibility of providing daily and on-demand PrEP to adolescent boys and girls in the three countries (South Africa, Uganda, and Zimbabwe) [part of the Combined HIV Adolescent PrEP and Prevention (CHAPS) study] [19]	Twenty-four group discussions and sixty in-depth interviews were conducted with males and females aged 13–24 years in Uganda, Zimbabwe, and South Africa. At the time of the study, Uganda had not rolled out PrEP to the general population, in Zimbabwe PrEP for AYP was only available at selected sites (one site within the study recruitment area), and for South Africa PrEP was available to selected high-risk groups. Most participants were PrEP-naïve, and PrEP was not readily available to young people in the three countries. Most participants received information about PrEP for the first-time during mobilization and implementation of this research	2018–2019	South Africa, Uganda, Zimbabwe	**Acceptability**: Young people expressed a willingness to use PrEP and identified potential barriers and facilitators of PrEP uptake. Barriers: Individual (fear of HIV, fear of side effects, and PrEP characteristics), interpersonal (parental influence, absence of a sexual partner), community (peer influence, social stigma), institutional (long waiting times at clinics, attitudes of health workers), and structural (cost of PrEP and mode of administration, accessibility concerns). Facilitators: individual (high HIV risk perception and preventing HIV), interpersonal (peer influence, social support and care for PrEP uptake), community (adequate PrEP information and sensitization, evidence of PrEP efficacy and safety), institutional (convenient and responsive services, provision of appropriate and sufficiently resourced services), and structural (access and availability of PrEP, cost of PrEP).
Informing the implementation of pharmacy-based PrEP for AGYW in Kenya by investigating experiences with pharmacy-based PrEP among AGYW seeking contraception at select retail pharmacies [32]	PrEP delivery was piloted at three retail pharmacies in Kisumu. AGYW purchasing contraception were offered PrEP by nurses with remote prescriber oversight. AGYW who accepted were provided with a free one-month supply. In-depth interviews were conducted with AGYW thirty days after receiving PrEP for qualitative analysis	2020–2021	Kenya	**Acceptability**: AGYW preferred pharmacies for accessing PrEP and they were willing to pay for PrEP even if available for free at clinics. Reasons for this preference included accessibility (pharmacies are more accessible than health facilities), lack of queues, and medication stockouts, privacy, anonymity, autonomy, and high-quality counselling (client-centred communication increases interest in PrEP) from study nurses.
A collaborative demonstration project of the first facility-based PrEP services for adults aged 18 years and older identifying as KP members [33]	The demonstration project introduced PrEP as a daily fixed-dose combination tablet of emtricitabine 200 mg-tenofovir disoproxil fumarate 300 mg and related services at four KP-friendly clinics in Kinshasa and three in Lubumbashi, for approximately 350 KP clients across all sites. PrEP was offered in combination with other HIV prevention services. Eligible clients were immediately initiated on PrEP, underwent screening and treatment for STIs, and received PrEP adherence and HIV risk-reduction counselling and condoms and lubricant	2018	Democratic Republic of Congo	**Reach**: 469 eligible KP clients were offered oral daily PrEP. Among these clients, 352 (75.1%) accepted. Among the 117 declining PrEP, 58 (49.6%) indicated that they were not interested in daily medication, and 35 (29.9%) did not believe they needed PrEP. **Maintenance**: Overall, 64.5% of clients who started PrEP attended the one-month visit. The three-month visit was attended by 289 (82.1%) of 352 clients initiated. Among clients attending the three-month visit, 91 (31.5%) had missed the one-month visit and were reengaged through outreach. Among 168 (47.7%) clients who initiated PrEP at least six months before data abstraction began, 144 (85.8%) attended the six-month visit. Among them, nine (6.3%) had missed their three-month visit and five had missed both one-month and three-month visits. No client who initiated PrEP seroconverted.
The FIKIA Project which was designed to take traditionally facility-based HIV services into communities to expand service reach, acceptability and uptake [34]	The FIKIA Project worked with diverse KP communities to tailor HIV services to their needs and to pair healthcare workers with trained peer educators and expert client counsellors to expand uptake of community-based HIV testing and ART services	2016–2022	Tanzania	**Reach**: 1 831 441 HIV tests were conducted in community settings; of the 98,349 (5.4%) individuals with new HIV diagnoses, 89,640 (91.1%) initiated ART. KP: 203,233 received HIV tests, 28,830 (14.2%) received a new HIV diagnosis and 25 170 (87.3%) initiated ART at the point of diagnosis. **Adoption**: HIV testing increased by 1.6 times overall (2.3 times among KP), HIV diagnoses increased by 8.7 times (10.9 times among KP) and ART initiation at the point of diagnosis increased from 80.0% to 95.9% overall (from 69.6% to 94.9% among KP).
A systematic review and meta-analysis to determine the effect of community-based ART initiation on HIV treatment outcomes [35]	Four randomised controlled trials and four observational studies in a total of 11 196 PLWH. Five studies focused on general HIV populations, two targeted key populations, and one involved adolescent. Community ART initiation strategies varied, including community-based HIV testing combined with ART initiation at home or community venues. Five studies-maintained ART refills within the community, while four provided refills at health facilities. Although all studies were pragmatic, most provided additional resources. However, few studies reported on implementation outcomes	2013–2021	Haiti, Lesotho, Malawi, Nigeria, South Africa, Tanzania, Uganda	**Adoption**: All studies showed higher ART uptake in community initiation arms compared to facility initiation and refill arms (standard of care) (RR 1.73, 95% CI 1.22 to 2.45; RD 30%, 95% CI 10% to 50%; 5 studies). **Effectiveness**: Retention (RR 1.43, 95% CI 1.32 to 1.54; RD 19%, 95% CI 11% to 28%; 4 studies) and viral suppression (RR 1.31, 95% CI 1.15 to 1.49; RD 15%, 95% CI 10% to 21%; 3 studies) at 12 months were also higher in the community-based ART initiation arms. Improved uptake, retention, and viral suppression with community ART initiation were seen across population subgroups—including men, adolescents, and key populations. One study reported no difference in retention and viral suppression at 2 years. Refill strategies following community ART initiation (community versus facility refills) found no difference in viral suppression (RD − 7%, 95% CI − 19% to 6%) or retention at 12 months (RD − 12%, 95% CI − 23% to 0.3%).
Assessment of the feasibility and acceptability of providing PrEP to AGYW (aged 16–25 years) via a community-based mobile health clinic—the Tutu Teen Truck (TTT) [40]	The TTT was originally developed in 2015 as an HIV testing and SRHS for adolescents and young people, geographically and logistically distinct from government health facilities’ day-to-day operations. The TTT integrated PrEP delivery into its provision of comprehensive sexual and reproductive health services. A qualitative exploration was undertaken with thirty young women	2017–2019	South Africa	**Adoption**: 585 young women started PrEP at the TTT between July 2017 – October 2019. **Acceptability**: A subset of 30 AGYW found the intervention for PrEP delivery as acceptable and accessible. The TTT provided services at times and in neighbourhood locations where AGYW organically congregate, thus facilitating service access and generating peer demand for PrEP uptake. The community-based nature and adolescent friendly health providers fostered a trusting provider-community-client relationship and strengthened AGYW HIV prevention self-efficacy. The integration of PrEP and sexual and reproductive health services delivery was highly valued by AGYW. While the TTT’s integration in the community facilitated acceptability of the PrEP delivery model, challenges faced by the broader community (community riots, violence and severe weather conditions) also at times interrupted PrEP delivery.
A qualitative study to understand the measures of continuity of HIV services (in accordance with the Ministry of Health guidelines for continuity of essential health services) during the COVID-19 pandemic restrictions in Kampala, Uganda [41]	Conducted at six large primary healthcare facilities in the Kampala Metropolitan area. Fourteen key informant interviews were performed lay health workers (*n* = 10) and ART focal persons (*n* = 4)	2021–2022	Uganda	**Adoption**: Leveraging the use of mobile phone technology for HIV care: Mobile-phone-based ART adherence support; mobile-phone-based psychosocial counselling; mobile-phone-based reminders about clinic appointments and referrals. Adoption of novel differentiated service delivery models for ART delivery: Use of motorcyclists to deliver ARVs; individualized ART delivery for patients with non-disclosed HIV status. Scale-up of existing differentiated service delivery models for ART delivery: Multi-month dispensing of ARVs; fast-track ARV refills; Home-based ARV refill; peer ART delivery approach; use of community pharmacy; continued use of community client-led ART delivery model. Reorientation of health facility functioning to the COVID-19 pandemic restrictions: Use of nearby health facilities for ARV refill and viral load monitoring; revised workflow; transportation of health workers to health facilities and flexible work schedules; introduction of shift work.
Sisters with a Voice (Sisters)—community-led differentiated HIV prevention and treatment services, including condoms, HIV testing, PrEP and ART linkage for sex workers [42]	PrEP service delivery was decentralized to community/home-based, peer-led PrEP services, to improve uptake and retention. New female sex worker PrEP initiations throughout 2020 were tracked by analysing routine Sisters programme data and comparing it with national PrEP initiation data for 2020. PrEP uptake among all negative FSW attending services in Sisters was mapped alongside Covid-19 adaptations and shifts in the operating environment throughout 2020	2020	Zimbabwe	**Reach**: 26 000 female sex workers across Zimbabwe in 2020. PrEP uptake in 2020 occurred at rates < 25% (315 initiations or fewer) per month prior to the emergence of Covid-19. In response to Covid-19 restrictions, differentiated service delivery models were scaled up in April 2020, including peer demand creation, community-based delivery, multi-month dispensing and the use of virtual platforms for appointment scheduling and post-PrEP initiation support. Beginning May 2020, PrEP uptake increased monthly, peaking at an initiation rate of 51% (*n* = 1360) in September 2020. Unplanned demand increases coincided with national commodity shortages between October and December 2020, resulting in new initiations being stopped and with sites prioritised refills.
Pharmacist-led PrEP programme (P-PrEP) comprised pharmacists from a university-based HIV clinic, a community pharmacy, and two community-based clinics. Through a collaborative practice agreement, pharmacists conducted PrEP visits with potential candidates for PrEP, according to the recommended guidelines, and authorized emtricitabine-tenofovir disoproxil fumarate prescriptions [43]	Demographics and retention in care over twelve months were summarised, and participant satisfaction and pharmacist acceptability with the P-PrEP programme were assessed by Likert-scale questionnaires	2017–2018	USA	**Adoption**: Sixty patients enroled in the P-PrEP programme between January and June 2017 completing 139 visits. **Maintenance**: Participant retention at 3, 6, 9, and 12 months was 73%, 58%, 43%, and 28%, respectively. **Effectiveness**: Zero seroconversions. **Acceptability**: All participants completing the patient satisfaction questionnaire would recommend the P-PrEP programme. Pharmacists reported feeling comfortable performing point-of-care testing and rarely reported feeling uncomfortable during PrEP visits (3 occasions, 2.2%) or experiencing workflow disruption (1 occasion, 0.7%).
Description of a collaboratively developed community pharmacy initiated HIV PrEP and PEP programme in San Francisco [44]	An interdisciplinary team of pharmacists, pharmacy personnel, public health physicians, and health department staff members worked together to design, launch, and maintain the programme. Pharmacists were trained on HIV testing and counselling and implementation of the PrEP protocol, including PEP initiation and STI testing. Patients' medical records were shared via a secure portal where physicians reviewed them and communicated with PrEP pharmacists as needed	2018–2019	USA	**Adoption/Reach**: Between April 2018 and the end of March 2019, 6 patients received PEP and 53 patients completed a PrEP initiation visit, of whom 96% (*n* = 51) filled their prescription.
A scoping review to describe evidence on the barriers to and facilitators of community pharmacy oral PrEP delivery, for pharmacists and pharmacy clients, using the capability, opportunity, motivation (COM-B) model [45]	Five bibliographic and five review databases were searched from inception to August 2023. Literature of any study design was included if it discussed barriers and facilitators of community pharmacy PrEP delivery. Trial registrations, protocols and news articles were excluded. Fifty-six met the inclusion criteria, predominantly from high-income/westernized settings. Most of the included literature was original research (55%), from the USA (77%) and conducted during or after the year 2020 (63%)	inception—2023	United Kingdom, USA, other	**Acceptability**: Barriers to PrEP delivery for pharmacists included lack of knowledge, training and skills (capability), not having the necessary facilities (opportunity), concern about the costs of PrEP and believing that PrEP use could lead to risk behaviours and sexually transmitted infections (motivation). Facilitators included staff training (capability), time, the right facilities (opportunity), believing PrEP could be a source of profit and could reduce new HIV acquisitions (motivation). For clients, barriers included a lack of PrEP awareness (capability), pharmacy facilities (opportunity) and not considering pharmacists as healthcare providers (motivation). Facilitators included awareness of PrEP and pharmacist’s training to deliver it (capability), the accessibility of pharmacies (opportunity) and having an interest in PrEP (motivation).
A review of novel platforms for biomedical HIV prevention delivery to KP outside of the traditional health facilities environment [46]	Community mobile clinics, peer-supported PrEP delivery, pharmacy-led PrEP delivery, and the mobile applications, telemedicine and home-based HIV prevention described and discussed	2013- 2021	Various including Kenya, South Africa, Tanzania, Thailand, USA, other	**Acceptability**: Mobile health clinics provide highly acceptable integrated, KP-focused services at convenient locations with the potential of high PrEP uptake. **Appropriateness**: Telemedicine and health apps decongest health systems and allow for personal agency and informed decision-making on personal health. Pharmacy-led PrEP delivery provides de-medicalised, confidential PrEP services at extended hours in community locations, from trusted medical professionals. Peer-supported delivery encourages continued PrEP use.
An survey of community pharmacists’ perceptions and attitudes regarding the supply of HIV medicines post ART inclusion in the Pharmaceutical Benefits Scheme (PBS) in 2016 [47]	A purposive sample of community pharmacists around Australia was invited to participate in an anonymous, online questionnaire that collected demographic information, and pharmacists’ perceptions and attitudes towards dispensing HIV, HBV, and HCV medicines for people living with HIV, HBC, and/or HCV	2016	Australia	**Appropriateness**: Of the 91 participants, the majority had not dispensed an HIV medicine. Most participants reported feeling comfortable when counselling people living with HIV about their medicines but a lack of confidence in knowledge of HIV and HIV medicines, and counselling patients about these medicines and managing the complex issues they experience. There was a statistically significant difference in genders—females reporting less confidence compared with males.
An assessment of the fidelity of a pharmacy-based oral HIV PrEP delivery model in five private pharmacies [48]	Model relied on a prescribing checklist with remote clinician oversight, which pharmacy providers used to identify clients unlikely to have any medical conditions that might contraindicate PrEP safety. Trained pharmacy providers delivered PrEP services, including identifying eligible clients, counselling on HIV risk, assessing PrEP safety, testing for HIV, and dispensing PrEP. Pharmacy clients completed surveys that assessed the fidelity of the services received after each visit. Standardized client actors were trained on four different case scripts, made unannounced pharmacy visits, and completed a 40-item checklist that assessed the fidelity and quality of service delivery components	2020—2021	Kenya	**Adoption**: From November 2020 to December 2021, 287 clients initiated and 159 (55%) refilled PrEP. **Fidelity**: At initiation, most clients were counselled on PrEP adherence (99%, 284 of 287) and potential side effects (97%, 279 of 287) and all received provider-assisted HIV self-testing before PrEP dispensing (findings consistent across refill visits). Nine standardized client actors completed 15 pharmacy visits. At each visit, most actors were asked about their behaviours associated with HIV risk (80%, 12/15) and all were counselled on PrEP safety and side effects. All actors reported that pharmacy providers treated them with respect.
A review to discuss the implementation challenges for the successful roll-out of CAB-LA in Africa and measures to address the implementation challenges [51]	Health system and client related challenges were elaborated on. Some solutions to the challenges were organised according to the sections of recommendations and demonstration projects, cost and availability of CAB-LA, cold chain issues, accessibility of CAB-LA, healthcare provider training, task shifting, community engagement and client education, and adherence promotion	2015–2022	Various including Kenya, South Africa, Uganda, other	Health system-level challenges included the cost of the drug, its refrigeration requirement, and the shortage of healthcare providers trained to administer parenteral medicines. Client challenges include lack of knowledge, accessibility of the drug, side effects, stigma, and lack of family and community support. Solutions can be adapted from the successful implementation of ART, oral PrEP, and immunization in Africa including advocating for waiving of CAB-LA patent licence, conducting demonstration projects in Africa, promoting the use of renewable energy sources, healthcare provider training, task shifting, community engagement, client education, and implementing adherence promotion strategies.
A scoping review of the published and grey literature to understand the potential for pharmacy-delivered HIV services in sub-Saharan Africa [53]	Using a modified Cochrane approach, electronic databases were searched through March 2022 and HIV conference abstracts in the previous 5 years for studies that: (1) focused on the delivery of HIV testing, antiretroviral therapy (ART) and/or PrEP at private pharmacies in sub-Saharan Africa; (2) reported on effectiveness outcomes (e.g. HIV incidence) or implementation outcomes, specifically feasibility and/or acceptability; and (3) were published in English	inception—2022	Various including Botswana, Ethiopia, Kenya, Nigeria South Africa, Uganda, other	Twenty-eight studies were included: seven on HIV testing, nine on ART delivery and 12 on PrEP delivery. Most studies (*n* = 16) were conducted in East Africa, primarily in Kenya. Two studies evaluated effectiveness outcomes; the majority (*n* = 26) reported on feasibility and/or acceptability outcomes. The limited **effectiveness** data (*n* = 2 randomized trials) suggest that pharmacy-delivered HIV services can increase demand and result in comparable clinical outcomes (e.g. viral load suppression) to standard-of-care clinic-based models. Studies assessing **implementation outcomes** found actual and hypothetical models of pharmacy-delivered HIV services to be largely **feasible** (e.g. high initiation and continuation) and **acceptable** (e.g. preferable to facility-based models and high willingness to pay/provide) among stakeholders, providers and clients. Potential barriers to implementation included a lack of pharmacy provider training on HIV service delivery, costs to clients and providers, and perceived low quality of care.
An assessment of the role/services community pharmacists play/offer in the management of HIV patients in Southern region of Nigeria, and the perception of community pharmacists towards management of HIV infected patients [54]	A cross-sectional study was carried out among 374 community pharmacist owned pharmacies spread across Rivers State using a structured questionnaire asking questions on demographic information, services offered to HIV infected patients and perception towards community pharmacists’ involvement in management of HIV	not listed—likely 2020	Nigeria	Out of 374 (100%) community pharmacist-owned pharmacies only 30 (8%) were involved in the management of PLWH. 23(76.6%) had special HIV training while 3(10%) self-acquired information on HIV. **Appropriateness**: The Likert scale to measure perception is biased towards agreement (score = 3.89), that community pharmacists are knowledgeable and well positioned to play role in the management of PLWH.
Assessment pharmacists’ knowledge of HIV/AIDS, perception towards the changing of treatment regimens of HIV/AIDS, attitudes towards PLWH and factors affecting the pharmacists’ attitudes towards PLWH [55]	A validated online forty-three item questionnaire was distributed to 400 pharmacists in Kelantan. Multivariate logistic regression was performed to identify factors associated with pharmacists’ negative attitude towards PLWH	not listed—likely 2019	Malaysia	One hundred and seventy respondents (42%) completed the questionnaire. Respondents had knowledge on the potential causes of HIV infection [median (IQR) score = 13.00(1); maximum score = 14.00], and preventive measures of HIV transmission [median (IQR) score = 12.00(1); maximum score = 12.00]. Respondents agreed on the need to change the HIV treatment regimen when required [median (IQR) score = 7.00(2); maximum score = 8.00]. Up to 40.6% thought that the treatment regimen should not be switched based on cost. Two-thirds of the respondents had negative attitudes towards PLWH (67.1%). Respondents who worked in community pharmacy reported positive attitudes towards PLWH (AOR = 0.125; 95%CI = 0.025–0.623; *p* = 0.011).
Feasibility assessment of integrating the community pharmacy as a task shift structure for differentiated community ART [56]	Stable patients on first line ART regimens from public health facilities were referred to community pharmacies in different locations within Abuja for prescription refills and treatment maintenance. Bio-demographic and clinical data were collected and analysed descriptively. The outcomes of measure were prescription refill and patient retention in care at the community pharmacy	2016–2017	Nigeria	**Adoption**: Approximately 10% of stable patients on ART were successfully devolved from eight health facilities to ten community pharmacies. **Maintenance**: Prescription refill was 100% and almost all the participants (99.3%) were retained in care after they were devolved to the community pharmacies. Only one participant was lost-to-follow-up because of death.
Feasibility, acceptability and outcomes of a community pharmacy-based ART refill model. Stable clients were referred to community pharmacies for routine refills at a service fee, to promote private sector participation and sustainability of ART services [57]	A population-based retrospective analysis of the community pharmacy ART refill programme was conducted	2016–2018	Nigeria	**Adoption**: Ten thousand and fifteen participants representing 14.4% of ART clients in 50 hospitals opted for this model and were devolved to 244 community pharmacies. All clients consented and paid a service fee of approximately $3 per refill visit. **Maintenance**: Median follow-up duration was 6 months. Prescription refill rate was 95% (95% CI 94.2–95.3). Retention rate was 98%. **Effectiveness**: Viral suppression was 99.12%.
Effectiveness evaluation of community-based private sector pharmacies provision of ART by comparing the CD4 cell count, weight and viral load of stable HIV patients before and after they were devolved to community pharmacies [58]	A facility-based retrospective study that analysed the data of PLWH accessing care at the University Teaching Hospital who were devolved to community pharmacies. Mean CD4 cell count, weight and viral load before they were devolved and 1 year after devolvement were compared	2018–2021	Nigeria	One hundred and seventy-one patients who met the eligibility criteria were down referred to community pharmacies during the study period. **Effectiveness**: Mean CD4 cell count (*p* = 0.001) and weight (*p* = 0.006) were higher after down referral to community pharmacies compared to when they were at the clinic. They all maintained viral suppression after being down referred.
A care pathway for PrEP delivery (ClinicalTrials.gov NCT04558554) was piloted in selected retail pharmacies [59]	Model used a prescribing checklist with remote clinician oversight, which pharmacy providers used to identify clients unlikely to have any medical conditions that might contra-indicate PrEP safety. Trained pharmacy providers delivered PrEP services, including identifying eligible clients, counselling on HIV risk, assessing PrEP safety, testing for HIV, and dispensing PrEP. Clients who did not meet the checklist criteria were referred to public facilities for free services delivered by clinicians. Pharmacy providers dispensed a one-month PrEP supply at initiation and a three-month supply thereafter at a client fee of ∼$3 USD per visit	2020–2021	Kenya	**Reach**: Pharmacy providers screened 575 clients, identified 476 who met the prescribing checklist criteria and initiated 287 (60%) on PrEP. **Maintenance**: PrEP continuation among clients was 53% (153/287) at 1 month, 36% (103/287) at 4 months and 21% (51/242) at 7 months. During the pilot, 21% (61/287) stopped and restarted PrEP and overall pill coverage was 40% (IQR 10%–70%). **Acceptability**: ≥ 96% of PrEP clients agreed or strongly agreed with statements regarding the acceptability and appropriateness of pharmacy-delivered PrEP services.
Evaluation of PrEP uptake, initiation, and continuation within a nurse-facilitated pharmacy-based delivery model for Kenyan AGYW seeking contraception at retail pharmacies [60]	PrEP-trained nurses were stationed at three retail pharmacies in Kisumu. AGYW purchasing contraception (emergency, oral contraceptive pills, injectables, implants, condoms) were counselled on PrEP, completed HIV testing, and offered a free one-month supply of PrEP pills per national guidelines by nurses under supervision of a remote physician. Evaluations were done of uptake among all AGYW offered PrEP and at 30 days after uptake PrEP use initiation and plans for continuation	2020–2021	Kenya	**Reach**: 85% of AGYW participants seeking contraception at retail pharmacies accepted daily oral PrEP when offered and 69% of those who initiated PrEP were willing to purchase it at retail pharmacies (median KES 150, IQR 100–200) even if it is available for free in public clinics.

* where dates were not provided the references were used as a proxy; ** where not provided the references were used as a proxy; *** where no clear alignment exists, results included as reported in literature

*USA* United States of America, *PrEP* preexposure prophylaxis, *STI* sexually transmitted infection, *HIV* human immunodeficiency virus, *ART* antiretroviral therapy, *LMIC* low- and- middle- income counties, *KP* key population, *AGYW* adolescent girl and young women, *PLWH* people living with HIV, *RR* risk ratio, *CI* confidence interval, *RD* risk difference, *ARV* antiretroviral, *HBV* hepatitis B virus, *HCV* hepatitis C virus, *CAB-LA* long acting injectable cabotegravir, *IQR* interquartile range, *AOR* adjusted odds ratio, *p* p-value, *USD* United States dollar, *KES* Kenyan shilling

## Challenges

Crawford et al. [63] identify policy, pharmacy, pharmacist and patient levels obstacles that could compromise the success of HIV mitigation amongst poor and rural populations. These populations are not dissimilar to those in LMICs. Addressing the first three levels involve putting measures in place to allow pharmacies to deliver and bill for PrEP services and promote pharmacies as legitimate PrEP providers. Pharmacies need to provide private spaces, and this should be complemented by pharmacists being trained in treatment protocols. Pharmacists should engage in continuous up-skilling particularly in providing HIV medication and should form referral networks. Patients particularly those who have problems accessing HIV medication, need to be educated about HIV to normalise accessing pharmacy services [63].

If these gaps occur in a high-income country, it is very likely that LMICs which have fewer resources will experience the same challenges but to a greater extent. Suphanchaimat R et al.’s. [64] *“Conceptual framework of the review on HIV/AIDS-related problems and services in low- and middle-income countries”* illustrates that epidemic burdens affect the access to services and vice versa [64]. The same holds true for the relationship between access to services and health systems management. These dynamics provide a less ideal picture of the achievability of the goals mentioned, simply because the operation of health structures in LMICs fall short of matching the pace of their high-income country counterparts.

LMICs do not have sufficient funds to carry out every project optimally. Dybul et al. assert that LMICs now bear the burden of providing over 50% of HIV funding as opposed to a decade ago where funding was provided largely by other wealthier international and global sources [65]. As a result of the reduction in PEPFAR support, it was reported that South Africa and Nigeria’s HIV management projects are becoming increasingly difficult to sustain, with Nigeria having less success in gathering domestic funding since their economy was weaker than South Africa’s [66]. In Uganda too, severe compromises had to be made in HIV treatment in response to PEPFAR’s cutbacks [67].

Over and above the challenges mentioned by Crawford et. al, it would seem prudent when evaluating whether community pharmacies can have a favourable impact on the provision of HIV ART PrEP in LMICs, to consider the number of community pharmacies (real and/or projected) within a country, and whether they are geographically dispersed enough to extend the roll out of HIV medication to larger numbers of the population [63]. In South Africa, there are 3580 community pharmacies spread out across the country. However, there still remains a conglomeration of them in urban areas, and fewer of them in rural locations, like in Chile 90% of the country’s market are serviced by chain pharmacies located in urban settings [26, 68]. A survey of pharmacies serving slum populations in Bangladesh, Nigeria, Kenya, and Pakistan reported that while accessibility to users was not an issue, there was variability in quality of service and prices [69]. This implies that while more people can indeed get access to their HIV medicines, a deliberate plan needs to be formulated to extend the services in hard-to-reach locations while still maintaining quality and affordability.

## Conclusion

Utilising community pharmacies and novel pick-up points for HIV PrEP in LMICs is still in its early stages of development, and there is much to address before the associated hurdles can be overcome, at an individual country level. Nonetheless, leveraging of learnings from HIV treatment programmes and formative PrEP initiatives it is evident that community pharmacies and pick-up points are inventive service points for at risk people and people with HIV. Reframing of health and governance policies with regards to both funding and general regulation, improving HIV medication and management skills of community pharmacists, formulating strategies to normalise HIV prevention and treatment at community pharmacies, and experimenting with innovative methods to develop patient centred access to medication, will be needed to scale up access to PrEP.

## Data Availability

No datasets were generated or analysed during the current study.
